# The Hospital Officers Association

**Published:** 1911-11-11

**Authors:** 


### The Hospital Officers Association.

### ANNUAL DINNER.

THE ninth annual dinner of the above Association took place on Saturday evening last at the Holborn Restaurant. The company, which numbered nearly ninety, sat down under the presidency of Mr. Richard Kershaw. In proposing the toast of the Association, the Chairman outlined the work that had been done by the Association and how its members met together and discussed different aspects and problems which accrued from time to time in connection with their particular institutions. He pointed out that prior to the establishment of the Association hospital workers were for the most part unknown to each other, and matters of interest were jealously guarded, whereas now they were discussed broadcast amongst the members, the result of which was general improvement all round. He (the Chairman) went on to say that some misapprehension seemed to exist as to what the Association really was. He wished to make it perfectly clear that it was established purely for the general discussion of hospital and institutional matters and social intercourse only, and in no sense was it a protection society for hospital employés.

Mr. W. T. Patrick, J.P., Mayor of Guildford, Secretary of Guildford Hospital, gave the toast "Our Hospitals," with which he incorporated the health of the guest of the evening, Colonel Sir John Young, C.V.O. Sir John Young, in responding, cited several interesting stories in connection with his career as a member of the Royal Army Medical Corps. His interview with Florence Nightingale, subsequent to his return from the Russo-Turkish War in particular made an entertaining story, and was a testimonial to her great thoughtfulness for others. The toast of "The Visitors" was proposed by Mr. E. Stewart Johnson, Secretary of the Hospital for Sick Children, Great Ormond Street, and was responded to by Dr. J. Dundas Grant, surgeon to the Central London Throat, Nose, and Ear Hospital, Gray's Inn Road, and Surgeon-General Evatt, C.B. Mr. Burleigh gave the final toast, "The Chairman." The musical direction was under the control of Mr. Robert Anthony. Mr. Dean, Secretary of Lord Mayor Treloar's Cripple Children's Fund, was at the piano and proved himself a very capable accompanist.

